# Aberrant neural representation of food stimuli in women with acute anorexia nervosa predicts treatment outcome and is improved in weight restored individuals

**DOI:** 10.1038/s41398-021-01630-1

**Published:** 2021-10-16

**Authors:** Ilka Boehm, Holger Mohr, Joseph A. King, Julius Steding, Daniel Geisler, Marie-Louis Wronski, Katharina Weigel, Veit Roessner, Hannes Ruge, Stefan Ehrlich

**Affiliations:** 1 Division of Psychological and Social Medicine and Developmental Neurosciences, Translational Developmental Neuroscience Section, Technische Universität Dresden, University Hospital C.G. Carus, Dresden, Germany; 2 Department of Psychology, Technische Universität Dresden, Dresden, Germany; 3 Department of Child and Adolescent Psychiatry, Eating Disorder Treatment and Research Center, Technische Universität Dresden, Faculty of Medicine, University Hospital C. G. Carus, Dresden, Germany

**Keywords:** Psychiatric disorders, Human behaviour

## Abstract

Anorexia nervosa (AN) has been associated with altered reward processing. We recently reported greater neural response in secondary visual areas when processing visual food stimuli in acutely underweight AN patients (acAN). In order to examine whether the observed alterations are indicative of acute undernutrition or a potential trait marker of AN, we set out to assess neural responses in acAN and in individuals weight-recovered from AN (recAN). FMRI data were collected from a total of 126 female volunteers, 35 acAN, 33 recAN, and 58 age-matched healthy controls (HC) while they viewed streams of food, social and neutral stimuli. A standard general linear model (GLM) was used to interrogate neural responses to the different stimuli in recAN vs. age-matched HC. Moreover, within-subject multivoxel pattern analyses (MVPA) in the two matched samples (acAN/HC and recAN/HC) were used to estimate neural representation of food vs. neutral, and social vs. neutral stimuli. A multiple regression analysis was conducted to test associations between the accuracy of the neural representation and treatment outcome. The GLM revealed no group differences between recAN and HC. The MVPAs showed greater classification accuracy of food stimuli in the posterior fusiform gyrus in acAN but not recAN. Classification accuracy was associated with better treatment outcome. Our findings suggest that the neural representation of food stimuli is altered in secondary visual areas in acAN and normalizes with weight recovery. Possibly this altered representation reflects attentional engagement motivating food intake, which may promote the recovery process.

## Introduction

Anorexia nervosa (AN) is defined by a restriction of food intake (or compensatory actions) driven by an intense fear of weight gain, which leads to dangerously low body weight [1]. AN is also associated with anhedonia [2, 3], the inability to experience pleasure in situations people usually enjoy. These phenomena have sparked the interest in dysfunctional reward processing in AN.

Previous neuroimaging studies targeting reward processing in AN have mostly applied food- and taste-related stimuli and pointed to alterations in the motivational striatal-insular brain circuit and prefrontal-cingulate areas associated with cognitive control [4–7]. However, available findings remain inconclusive, particularly regarding the exact location and direction of changes in neural responses to food or other rewarding stimuli [5]. The current evidence is also limited and heterogeneous regarding the question whether neural alterations persist in individuals with a history of AN after weight- restoration and symptom remission (recAN). While some results suggest that hypoactivation in motivational brain regions is also evident in recAN [8], others suggest altered responses in brain regions associated with motivation and cognitive control present during the acute phase of AN (acAN) do not persist into recovery [9]. In addition to the large literature focused on reward-related processing associated with body perception [10–14] applying positive social stimuli is a potentially informative means of studying a nondisorder specific, but nonetheless for general mental health highly relevant stimulus domain. However, there is a scarcity of research regarding neural responses to social reward in AN. First results suggest altered response in frontal and parietal brain regions [15–17]. Results from behavioral studies show reduced responses to pleasant social stimuli [18] such as funny film clips [19, 20], pleasant touch [21], and pleasant female faces [22] in patients with AN. In our own previous work [23], we aimed to disentangle the relative contribution of the brain reward and cognitive control systems by presenting rewarding food, social and neutral stimuli in a subliminal and supraliminal fashion. Subliminal stimuli are usually processed with no or little awareness and are therefore less likely to provoke a robust fronto-parietal response—thus impeding a modulating influence of the cognitive control system on reward-related brain regions. While no group differences in neural activity were detected in the subliminal condition, patients acutely ill with AN (acAN) exhibited generally elevated activation in the inferior frontal junction, a brain region associated with cognitive control [24], during supraliminal stimulation regardless of stimulus type. Moreover, patients exhibited increased activation when processing food pictures in regions of visual cortex including the fusiform gyrus (FG). Our previous study focused on acutely underweight patients and did not allow to draw any conclusions on whether the findings represent a marker of undernutrition or a biological vulnerability towards AN.

Even though only large longitudinal studies can provide ultimate answers, an approach that may help to distinguish state from trait markers may be the inclusion of patients recovered from AN (recAN). Recent fMRI studies have investigated the neural representations of various stimuli to answer not only the question as to where certain stimuli are processed in the brain, but also how they are represented. Neural representations can be identified using multivariate-pattern analysis (MVPA), an approach for decoding stimulus categories or experimental conditions from neural activations. In contrast to the mass-univariate approach, MVPA integrates information from multiple voxels into a single model, thus allowing to analyze patterns of neural activation instead of voxelwise and, thus, solely localized activation differences [25]. Since complex phenomena such as mental disorders are likely to be underpinned by overarching neurophysiological alterations, MVPA seems to be a particularly valuable statistical approach in this field [26].

### Purpose of the present study

In an attempt to shed new light on the question whether neural alterations during the passive viewing of rewarding stimuli are a state or trait marker of AN, we examined both acAN and recAN in comparison to HC. First, we implemented the analysis approach laid out in Boehm et al. [23] using fMRI data obtained in recAN compared to age-matched healthy control (HC). Since alterations during reward processing are suggested to be a risk factor of AN we assumed that recAN would show similar neural alterations in prefrontal and secondary visual areas as previously reported acAN [23]. Second, taking advantage of recent methodological developments, we applied MVPA to fMRI data during supraliminal stimulus presentation of acAN, recAN, and HC in order to make inferences about the neural representations underlying reward processing.

## Method

### Sample

The sample of the current study consisted of a total of 126 female volunteers: 35 patients with acute AN according to DSM-5 (12–29 years old; same sample as in Boehm et al. [23]), 33 individuals weight-recovered from AN (15–29 years old; newly acquired sample; recAN), and 58 age-matched healthy controls (12–29 years old). Because of the difference in mean age (Table 1) between the two patient populations and possible associated developmental effects, two independent pairwise age-matched groups were formed—acAN/HC_acAN_ and recAN/HC_recAN_—by means of an automated search algorithm (see Supplemental Materials (SM) 1.1). All acAN were admitted to eating disorder programs of a university child and adolescent psychiatry and psychosomatic medicine department and were assessed within 96 h after beginning a behaviorally-oriented nutritional rehabilitation program. Diagnosis was supported using a for DSM-5 modified version of the Structured Interview for Anorexia and Bulimia Nervosa [27], which requires body mass index (BMI) below the 10th age percentile (if younger than 15.5 years) and below 17.5 (if older than 15.5 years). To be considered “recovered”, recAN subjects had to (1) maintain a BMI > 18.5 kg/m^2^ (if older than 18 years) or a BMI > 10th age percentile (if younger than 18 years) for at least 6 months prior to the study, (2) menstruate, and (3) have not binged, purged, or engaged in significant restrictive eating patterns. HC participants had to be of normal weight (BMI > 18.5 kg/m^2^) and eumenorrhoeic and without any history of psychiatric illness (SM 1.1). HC were recruited through advertisement among middle school, high school and university students. We applied several additional exclusion criteria for each group (SM 1.1)—most importantly psychotropic medication (other than SSRI; acAN *n* = 1, recAN *n* = 3) within 4 weeks prior to the study, binge eating, or a diagnosis of bulimia nervosa, substance abuse, neurologic, or medical conditions. This study was approved by the Institutional Review Board of TU Dresden, and all participants (and if underage their guardians) gave written informed consent.

**Table 1:** Means and standard deviations for demographic and clinical characteristics.

	Sample I		Statistics		Sample II		Statistics	
	acAN	HC_acAN_	*T*	*p*	*recAN*	*HC*_*recAN*_	T	*p*
Age	16.20 ± 3.46	16.29 ± 3.32	−0.116	0.908	22.22 ± 3.51	21.83 ± 3.56	0.45	0.652
BMI-SDS	−3.191 ± 1.18	0.039 ± 0.65	−14.19	<0.001	−0.55 ± 0.60	−0.30 ± 0.60	−1.75	0.084
BMI	14.62 ± 1.50	20.74 ± 2.21	−13.54	<0.001	20.64 ± 1.66	21.34 ± 1.82	−1.65	0.104
EDI-2 total	213.46 ± 42.90	145.61 ± 30.06	7.66	<0.001	179.23 ± 55.58	133.12 ± 22.54	4.40	<0.001
BDI-II	23.42 ± 9 .71	5.98 ± 6.64	8.77	<0.001	10.18 ± 9.94	3.67 ± 4.56	3.42	0.001
Hunger	4.52 ± 2.58	6.05 ± 2.39	−1.94	0.06	5.43 ± 2.14	6.50 ± 2.22	1.34	0.19
MRS	8.00 ± 2.35	n.a.			n.a.	n.a.	n.a.	n.a
Duration of illness (in month)	12.8 8 ± 19.4	n.a					n.a	n.a
Duration of recovery (in month)					59.30 ± 55.79)			

*acAN* acute anorexia nervosa patients, *recAN* recovered anorexia nervosa patients, *HC*_*acAN*_ healthy subsample age-matched to acute anorexia nervosa patients, HC_recAN_ healthy subsample age-matched to recovered anorexia nervosa patients, *BMI-SDS* body mass index standard deviation score, *BMI* body mass index, *EDI-2 total* eating disorder inventory-2 total score, *BDI-II* Beck depression inventory-II, *MRS* Morgan-Russell outcome score.

### Instrument

For all participants, current diagnoses of eating disorders were ascertained by evaluation of the expert form of the Structured Interview for Anorexia and Bulimia nervosa (SIAB-EX) [27]. For more details, see SM 1.2 and Supplementary Table 1. Eating disorder-specific psychopathology was assessed with the short version of the Eating Disorder Inventory-2 (EDI-2) [28]. Depressive symptoms were evaluated using Beck Depression Inventory (BDI-II) [29]. Self-reported appetite, including the subscale “hunger”, was measured with the use of a visual analogue scale ranging from 0 (no hunger) to 10 (extreme hunger) [30]. Treatment outcome after 1 year was assessed using the Morgan-Russell outcome assessment scale [31]. Due to the young age of the participants, we calculated the mean score of the subscales ‘food intake’, ‘menstrual state’, ‘mental state’, and ‘socioeconomic state’ and excluded the subscale ‘psycho-sexual state’.

### Task

As reported previously, participants viewed streams of food stimuli, social stimuli, and neutral stimuli presented either subliminally or supraliminally while fMRI data were obtained [23]. Details regarding the selection of visual stimuli and results of the independent pilot testing of the stimuli are described in SM 1.3. Most importantly, as intended, mean arousal ratings for neutral stimuli (mean 2.6(SD 0.5)) were indeed significantly lower on a nine-point likert scale than those for social stimuli (mean 4.5 (SD 0.9) (T(44.24) = 10.64; *p* < 0.001). The paradigm was divided into four equally long blocks in which the first two involved subliminal stimulation and the last two supraliminal stimulation. Subliminal blocks were shown first, as familiarity of the stimuli may promote conscious processing even under subliminal stimulation condition [32]. Each long block consisted of nine mini-blocks (three of each stimulus category in pseudo-randomized order), each composed of 10 trials. In the subliminal trials, the stimuli were presented for 17 ms, followed by a mask for 150 ms (backward masking) and a crosshair (fixation) for 1309 ms. In each supraliminal trial, a stimulus was presented for 500 ms, followed by a crosshair presented for 973 ms. Thirty neutral and thirty social stimuli were selected from the International Affective Picture System (IAPS) [33] and the database EmoPics [34]. The 30 food stimuli employed in this study originated from a dataset by Kroemer et al. [35]. For more details on stimulus selection, please refer to SM 1.3. Each stimulus was presented four times, i.e., once in each subliminal and supraliminal long block.

### Structural and functional image acquisition

Data was acquired between 8 and 9 AM after an overnight fast using a standard 3 T Siemens Trio, equipped with a standard 12 channel head coil. T1-weighted structural brain scans were acquired with rapid acquisition gradient-echo (MP-RAGE) sequence with the following parameters: number of slices = 176; repetition time = 1900 ms; echo time = 2.26 ms; flip angle = 9°; slice thickness = 1 mm; voxel size = 1 × 1 × 1mm³; field-of-view = 256 × 224 mm²; bandwidth = 2004 Hz/pixel. Functional images were acquired by using a gradient-echo T2*-weighted echo planar imaging with the following parameters: tilted 30° towards AC–PC line (to reduce signal dropout in orbitofrontal regions); number of volumes = 190; number of slices = 40; repetition time = 2410 ms; echo time = 25 ms; flip angle of 80°; 3.4 mm in-plane resolution; slice thickness of 2 mm (1 mm gap resulting in a voxel size of 3 × 3 × 2 mm); FoV=192x192mm; bandwidth of 2112 Hz/pixel.

### Image data preprocessing

Functional and structural images were processed using the SPM8 toolbox (http://www.fil.ion.ucl.ac.uk/spm/) within the Nipype framework (http://nipy.sourceforge.net/nipype/). The slice time-corrected functional data were realigned and registered to their mean. The realigned files were coregistered to the subject’s structural brain image. A DARTEL template was created using structural images from all subjects. The EPI volumes were then normalized to MNI space using the DARTEL template and corresponding flow field. For the general linear model (GLM) analysis (mass-univariate), the resulting data were smoothed with an isotropic 8 mm FWHM Gaussian kernel while no smoothing was applied for the multivariate-pattern analysis (MVPA; see below) at the single-subject level [36].

We evaluated the quality of the fMRI data by manual inspection and using artifact detection tools (ART) [37]. Volumes that exceeded an intensity threshold of three standard deviations or a threshold of 2 mm normalized movement in any direction were classified as outliers. The groups did not differ significantly regarding intensity and motion outliers. For more details, refer to SM 1.4 and Supplementary table 2.

### Analysis using the general linear model

The GLM was setup as in our previous study [23]. Briefly, on the 1st level, a GLM was fitted to the hemodynamic response to each of the six combinations of stimulation conditions and stimulus types: supraliminal food stimuli, subliminal food stimuli, supraliminal social stimuli, subliminal social stimuli, supraliminal neutral stimuli, and subliminal neutral stimuli using boxcar functions with a duration of 15 s (epoch-related design). Additionally, six realignment parameters and outlier volumes identified by quality control as described above were included as nuisance regressors. On the 2nd level, a linear mixed model including a two-level within-subject variable (stimulation condition: supraliminal, subliminal), a three-level within-subject variable (stimulus type: food, social, neutral), and a binary between-subject variable (group: recAN, HC_recAN_) was estimated using the GLMflex toolbox (http://mrtools.mgh.harvard.edu/index.php/GLM_Flex). To address our main research question regarding differences between the groups when processing the different stimulus types under the two stimulation conditions, we examined the group × stimulation condition × stimulus *type* interaction. To guard against type I errors, the interaction had to exceed a significance level of *p* < 0.05 family-wise error rate corrected (cluster-level).

### Analysis using multivoxel pattern analyses

In attempt to minimize potentially confounding developmental effects (given that recAN are systematically older than acAN), we conducted two separate (but identical) analyses of pairwise age-matched case-control between-group comparisons: (1) A group comparison between acAN vs. HC_acAN_ (*n* = 35 in each group; referred to as acAN/HC_acAN_ sample), and (2) a group comparison between recAN vs. HC_recAN_ (*n* = 33 in each group; referred to as recAN/HC_recAN_ sample). Since our previous findings in acAN [23] and the current mass-univariate analysis in recAN revealed no group difference in processing subliminally presented stimuli (see Results), we applied the MVPA only to the supraliminal presentation condition.

At the single-subject level, a GLM was fitted voxelwise to the hemodynamic responses using a block design. Different from the mass-univariate approach, a separate regressor was included for each mini-block, resulting in 18 block regressors overall. Additionally, six realignment parameters and outlier volumes identified by quality control as described above were included as nuisance regressors.

The voxel-specific BOLD estimates of the 18 mini-blocks were then used as input for two different within-subject MVPAs. In the first MVPA, a linear support vector machine (SVM) was trained to predict food vs. neutral stimuli, and in the second MVPA, the SVM was trained to predict social vs. neutral stimuli. The Decoding Toolbox (TDT) [38] was used to implement a searchlight analysis with a radius of 12 mm and fixed SVM hyperparameter C = 1 using a leave-two-mini-blocks-out cross-validation scheme. In each cross-validation fold, the SVM was trained on 10 mini-blocks (5 from each condition) and tested on the left-out two mini-blocks (1 from each condition) resulting in 6 × 6 = 36 cross-validation folds. This procedure resulted in two whole-brain accuracy maps (corresponding to the classification accuracies for food vs. neutral and social vs. neutral stimuli) per subject.

For group-level analyses, the individual accuracy maps were smoothed using a Gaussian kernel of 8 mm FWHM and submitted to SPM8. First, we evaluated in which brain regions the classifier could differentiate above chance level between the different experimental conditions using one-sample *t*-tests across both groups within each sample individually. Second, the smoothed accuracy maps were submitted to two-sample *t*-tests to investigate whether patients had a different neural representation of food or social stimuli vs. neutral stimuli in comparison to HC in whole brain (separately for the acAN/HC_acAN_ and recAN/HC_recAN_ sample). Between-group differences had to exceed a significance level of *p* < 0.05 family-wise error corrected to guard against type I errors (FWE correction at the cluster-level; cluster-defining threshold *p* = 0.001). Given known structural alterations in AN [39], we inspected potential structure-function relationships by including cortical thickness measures of the identified cluster as a covariate in the group-level analyses. For more details, refer to SM 1.5.

In order to examine whether altered neural representation in the posterior FG represents a helpful neural adaption process to the state of undernutrition (see Discussion), we extracted the β-values of the cluster of the posterior FG and ran a correlation analysis with BMI-SDS and a post-hoc multiple linear regression analysis with classification accuracy at posterior FG and BMI-SDS at date of fMRI data acquisition as predictors (simultaneously included in the model) and Morgan-Russell outcome scale as dependent variable.

## Results

### Sample characteristics

Demographics and clinical characteristics are summarized in Table 1. Neither acAN nor recAN differed regarding age to their respective HC counterparts (HC_acAN_ and HC_recAN_). As expected, acAN had a higher EDI-2 as well as BDI-II total score and lower BMI-SDS than HC_acAN_. Overall, despite weight normalization and generally improved psychopathology, recovered individuals showed some residual eating disorder and depressive symptoms. Treatment outcome after 1 year was gathered in 27 participants of the acAN group. Two patients had a poor outcome (Morgan-Russell outcome score < 4), 9 an intermediate outcome (score 4–8) and 16 a good treatment outcome (score > 8).

### Imaging results—GLM

As in our previous study in acAN, stimulus-specific activity was found in both the subliminal and supraliminal stimulation condition in the recAN/HC_recAN_ sample. Specifically, increased BOLD responses were observed in reward-related brain regions such as the ventral striatum for social vs. neutral stimuli types (see SM 2 and Supplementary Fig. 1).

In contrast to our previous results [23] where acAN showed a generally increased response in the inferior frontal junction and an increased activation in the FG/parahippocampal gyrus and cuneus during supraliminal stimulation with food stimuli, the mass-univariate approach revealed no significant group × stimulation condition × stimulus type interaction for the recAN/HC_recAN_ sample. To follow up, we investigated the supraliminal food > neutral and social > neutral contrasts within a restricted search space by applying a mask corresponding to the findings in acAN (FG/parahippocampal gyrus and cuneus, respectively [23]). Results revealed no group difference.

### Imaging results—MVPA

**Fig. 1:**
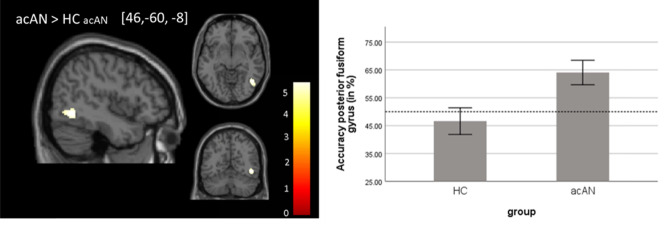
Group difference in classification accuracy between acAN and HC_acAN_ in the food vs. neutral contrast (*p* < 0.05, FWE); left panel: whole-brain map of the group difference; right panel: extracted classification accuracy values of the fusiform gyrus plotted for healthy control (HC) and acutely ill patients (acAN). Left panel: peak coordinate in brackets; right panel: dashed line marks the chance level; error bars represent 95% confidence interval.

Across groups, mean classification accuracies were above chance in secondary visual areas both for the classification of food vs. neutral and social vs. neutral stimuli. For more details, please refer to SM 3.1 and Supplementary Fig. 2. Group comparisons revealed that acAN were characterized by a higher decoding accuracy than HC_acAN_ for the classification of food vs. neutral stimuli within posterior FG (T = 5.65; *p* = 0.001 (FWE); [46; −60, −8]; k = 128), see Fig. 1. To ensure that group differences in decoding accuracy were not driven by known structural brain alterations in acAN [39], we controlled for cortical thickness of the FG (T = 6.50; *p* < 0.001 (FWE); [44; −58, −12]; k = 448; see Supplementary Fig. 3). The finding remained significant. Furthermore, the finding was also robust when patients with comorbid disorders (*n* = 3) were excluded (T = 5.40; *p* = 0.013 (FWE); [46; −60, −6]; k = 25; see Supplementary Fig. 4). No group differences between acAN and HC_acAN_ were found for the classification of social vs. neutral stimuli. The group comparisons between recAN and HC_recAN_ resulted in no significant differences for the classification of food vs. neutral and social vs. neutral stimuli. Restored neural representation of food stimuli in the recAN group was also suggested by the absence of significant group differences between recAN and HC_recAN_ when restricting the search space by applying a mask corresponding to the finding in acAN (cluster within posterior FG).

Post-hoc supplementary analysis using Bayesian independent samples *t*-test via JASP (https://jasp-stats.org/) were applied to further investigate the amount of evidence for the absence of group differences between recAN and HC_recAN_ within clusters found to be different between acAN and HC_acAN_, according to the MVPA approach (see SM 4 and Supplementary Table 3). Results showed moderate evidence for the null hypothesis indicating no difference between recAN and HC_recAN_.

Classification accuracy of the posterior FG was not associated with BMI-SDS at date of data acquisition in acAN. Post-hoc multiple linear regression analysis (all assumptions of the linear regression were met) indicated that the Morgan-Russell outcome 1 year after treatment of acAN could be predicted by classification accuracy at the FG and BMI-SDS at the day of fMRI acquisition (F(2,23) = 6.103; *p* = 0.007). Testing the individual predictors for statistical significance showed that both the classification accuracy at FG (β = 0.38; b = 0.077; CI_b_ = 0.008–0.147; T = 2.30; *p* = 0.031) and the BMI-SDS (β = 0.43; b = 0.91; CI_b_ = 0.191–1.628; T = 2.61; *p* = 0.015) contributed to the prediction.

## Discussion

The objective of this study was to elucidate whether alterations in the processing of food and social reward constitute a trait or state marker of AN. To this end, we studied acAN as well as former AN patients after weight recovery using fMRI data collected during viewing of rewarding stimuli and applied two analysis strategies: First, we employed a GLM method that we previously used in acAN [23], in a newly acquired recAN sample. Next, we applied an MVPA approach, which allowed to investigate how specific stimulus categories were represented in the brain in both samples.

Unlike in acAN, when applying the mass-univariate approach, neural responses to rewarding food and social stimuli were unaltered in the recAN group. Likewise, acAN but not recAN showed greater accuracy for the classification of food stimuli in secondary visual areas, namely the posterior FG, when applying MVPA. This suggests that not only the magnitude of the neural response to food stimuli but also the neural representation of food stimuli is altered in acAN and seems to normalize with weight recovery. Furthermore, accuracy for the classification of food stimuli in the FG in acAN individuals was associated with better treatment outcome after 1 year.

The (relative) normalization of brain states in recAN is in line with findings of Frank et al. [40] who used a multivariate approach for decoding of insula activity during taste perception. They found alterations in classification accuracy in patients suffering from obesity as well as in acAN but not recAN. Other previous studies in recAN have used conventional fMRI analysis methods: In a study focusing on insula-striatal circuits, recAN showed reduced neural response during sucrose application [41]. Likewise in a recent study by Kaye et al. [4], recAN showed reduced striatal response to sucrose solution. With respect to food pictures, previous work also found reduced neural response in the amygdala, hypothalamus, and anterior insula during the acute and recovered state of the disorder [8]. In contrast to this and in line with our findings, Scaife et al. [9] found no group difference between recAN and HC and between recAN and acAN in neural responses during the processing of food pictures, supporting our assumption that the abnormal neural response and representation of food-related stimuli in AN may be an adaptation to the state of undernutrition, which abates with weight restoration. The region of altered neural representation of food stimuli in this study—the FG—is part of the occipital and temporal lobe and a key structure for high-level visual processes, such as face-perception, reading, and object recognition [42].

The posterior FG has repeatedly been found to be a part of the neural network activated during the visual processing of food stimuli [43]. It has also been shown to be sensitive to motivational states (hunger and satiety) and related peripheral hormones such as ghrelin [35] and insulin [44]. However, different states of hunger are less likely to account for the group difference in this study as both groups fasted overnight. Other factors known to affect the functioning of the FG are body weight, respectively, body weight change and, related to it, the adipocyte-derived hormone leptin [45–47], which is important in energy homeostasis [48] and has been found to be associated with food-related thoughts in AN [49]. Previous work in obesity demonstrated that after weight loss—a condition of relative leptin deficiency—brain areas of the ventral visual stream including the FG show an increased neural response and functional connectivity to the hypothalamus, a region of homeostatic regulation, when processing food cues [45, 47]. Interestingly, this neural alteration was reversible with leptin administration [45]. As the acute state of AN is also characterized by leptin deficiency-, which normalizes with weight restoration [50], these findings support our assumption that the altered neural activity to and neural representation of food stimuli in the FG may constitute a state marker of the disorder.

Besides its implication in food processing, the right posterior FG has also been closely associated with representation and identification of emotionally relevant information. In a fMRI study by Bradley et al. [51], greater FG response was found in participants viewing arousing pictures. Therefore, it has been suggested that the visual system, in particular the right posterior FG, encodes differences in attentional engagement for emotionally relevant stimuli in order to direct appropriate behavioral responses. This interpretation dovetails with the above-discussed role of the FG as part of a network sensitive to homeostatic needs [35, 45]. In a condition of acute hunger or leptin deficiency, e.g., due to reduced body weight, food stimuli are of high emotional and behavioral relevance requiring immediate attention. From this, one might conclude that the distinct neural representation of food stimuli in the posterior FG in patients acutely ill with AN may reflect an adequate and healthy neural reaction to the state of starvation that should motivate food intake, which is supported by the predictive value of classification accuracy of the posterior FG for treatment outcome after one year. This is in line with findings by Frausto et al. [52] who reported increased neural activity in occipital-temporal brain regions in early time intervals during the processing of food stimuli as observed using magnetoencephalography.

Similar to our findings with food stimuli, neural responses to and classification accuracy of social stimuli were unaltered in recAN. Likewise, previous work found no difference between recAN and HC when watching happy faces [53, 54]. In contrast, McAdams et al. [15] found alterations in parietal brain regions in acAN as well as recAN in reaction to positive social feedback. This discrepancy may be explained by a stronger saliency of social feedback. Interestingly, previous nonimaging studies rather collectively report reduced social reward responsiveness in AN [18]. Studies investigating gaze pattern suggest that AN patients tend to avoid looking at face stimuli, which might have reduced a deeper neural processing and may partially explain the reported findings here [22, 55].

### Limitations

The interpretation of the study rests upon the following limitations: First, due to age differences we compared results obtained in acAN and recAN only qualitatively across studies and analyses but did not include them into one statistical model in order to limit confounding effects with age. Second, studying recAN allowed us to exclude the effects of acute undernutrition, but the cross-sectional nature of the study does not allow determining whether the detected differences constitute a true trait marker or rather a possible scar-effect of the disease [56]. Third, we cannot comment on how our findings relate to chronic or relapsing-remitting patients. Fourth, the standardized stimulus material was drawn from three different datasets. Although we carefully selected stimuli to be matched according to overall entropy and brightness while excluding potentially confounding pictures (e.g., erotic images), our stimulus material was drawn from three databases and future studies might strive to draw stimuli from larger standardized databases [11, 57].

Moreover, while the results of both the GLM and decoding approach suggest that food stimuli have a high saliency for AN, it remains unclear how patients manage to abstain from food intake. One explanation might be that acAN patients perceive food images as highly salient, but that the valence of the stimuli is negative, i.e., threatening or disgusting. Alternatively, the stimuli might have a positive valence, but patients engage in excessive self-control [23]. Future studies investigating the processing of food stimuli will benefit from the consideration of endocrinological parameters such as leptin and insulin. A strength of our study is the fact that all participants were scanned at the same time of the day after an overnight fast [58].

## Conclusion

Our study suggests that distinct neural representations of food stimuli in the right posterior FG, a brain region with a known role in the identification of highly salient stimuli, are present during the acute state of AN but were not detected after weight restoration. We suggest that altered representation of food in high-level visual areas constitutes a neural adaptation to the life-threatening state of undernutrition in AN patients and may help to direct attention towards and motivate for food intake and, ultimately, promote recovery. Although warranting replication, this study might send a positive message to patients, clinicians and caregivers that even in a severely ill state the neural system has the potential to adapt information processing to promote the rehabilitation process.

## Supplementary information

Supplementary material
