# Supplementary material for: Aberrant neural representation of food stimuli in women with acute anorexia nervosa predicts treatment outcome and is improved in weight restored individuals
Source: Transl Psychiatry. 2021 Oct 16;11:532. doi: 10.1038/s41398-021-01630-1 (PMC8520531; doi:10.1038/s41398-021-01630-1)
Supplement: Supplementary file 1 — Supplementary material [file 41398_2021_1630_MOESM1_ESM.doc]

**Supplemental Materials (SM)**

**SM 1 Method**

**SM 1.1 Sample**

Comorbid psychiatric diagnoses were made by an expert clinician and included the examination of the participant and careful archival chart review (including medical and psychiatric history, physical examination and several psychiatric screening instruments).

Exclusion criteria and possible confounding variables, e.g. the use of psychotropic medications and medical comorbidities, were obtained using the SIAB-EX ^1^ and our own semi-structured interview. Within the acAN group, 2 participants were of the binge/purge subtype and 3 participants had associated psychiatric comorbidity at the time of treatment (2 depressive disorders and 1 obsessive compulsive disorder). No acAN was taking antidepressant or any other psychotropic medication. Within the recAN group, 8 participants were of the binge/purge subtype and 6 participants had associated psychiatric comorbidity (5 depressive disorders including dysthymia and 1 obsessive compulsive disorder and 2 anxiety disorders) and 3 were taking antidepressants (SSRI) at the time of treatment. Comorbid psychiatric diagnoses were made by an expert clinician and included the examination of the participant and careful archival chart review (including medical and psychiatric history, physical examination and several psychiatric screening instruments).

HC participants were excluded if they had any history of psychiatric illness, a lifetime BMI below the 10^th^ age percentile (if younger than 18 years) or a BMI below 18.5kg/m^2^ (if older than 18 years), or if they were currently obese (BMI over 97^th^ age percentile if younger than 18 years; BMI over 28kg/m^2^ if older than 18 years).

Participants of all study groups were excluded if they had a lifetime history of any of the following clinical diagnoses: organic brain syndrome, schizophrenia, substance dependence, psychosis NOS, bipolar disorder, bulimia nervosa or binge-eating disorder (or “regular” binge eating - defined as bingeing at least once weekly for three or more consecutive months). Further exclusion criteria for all participants were IQ lower than 85; psychotropic medication within four weeks prior to the study; current substance abuse; current inflammatory, neurologic or metabolic illness; chronic medical or neurological illness that could affect appetite, eating behavior, or body weight (e.g., diabetes); clinical relevant anemia; pregnancy; breast feeding. Pairwise case-control age-matching was carried out using the Munkres algorithm ^2^. Maximum age distance between the pairs was 0.9 for the acAN vs. HC_acAN_ sample and 0.8 for the recAN vs. HC_recAN_ sample. Ten HC were included in both samples.

Study data were collected between May 2014 and September 2015 and managed using a secure, web-based electronic data capture tool (REDCap, Research Electronic Data Capture ^3^). The aim of this study was to investigate whether observed neural alterations found in Boehm et al.^4^ are also present at the weight-recovered state of the disorder. Therefore an equivalent sample size as in Boehm et al.^4^ was chosen.

**SM 1.2 Instruments**

For all participants, current diagnoses of eating disorders were evaluated by the expert form of the SIAB-EX ^1^, a well-validated 87-item semi-standardized interview that assesses the prevalence and severity of specific eating-related psychopathology over the past three months. The interview provides diagnoses according to the ICD-10 and DSM-IV. Interviews were conducted by clinically experienced and trained research assistants under the supervision of the attending child and adolescent psychiatrist.

Intelligence quotient (IQ) was assessed with a short version of the German adaptation of the Wechsler Adult Intelligence Scale ^5^ for participants aged 16 years and older or a short version of the German adaptation of the Wechsler Intelligence Scale for Children ^6^ for participants aged 15 years or younger.

**Supplementary table 1**

***Means, standard deviation and test statistics of EDI2 and BDI-II in both samples***

|  | *acAN* | *recAN* | *HC_recAN_* | *acAN vs. recAN* | | *recAN vs. HC_(recAN)_* | |
| --- | --- | --- | --- | --- | --- | --- | --- |
|  | *M (SD)* | *M (SD)* | *M (SD)* | *T* | *p* | *T* | *p* |
| *EDI2* | 213.46 (42.89) | 179.23 (55.58) | 133.12 (22.54) | -2.85 | 0.006 | 4.40 | <0.001 |
| *BDI-II* | 23.42 (9.94) | 210.18 (9.94) | 3.67 (4.56) | 05.55 | >0.001 | 3.42 | <0.001 |

**SM 1.3 Stimuli**

Thirty neutral and thirty social stimuli were selected from the International Affective Picture System (IAPS) ^7^ and the database EmoPics ^8^. Neutral stimuli showed e.g., plants or household items, while social stimuli showed happy social sceneries (e.g. playing children). The thirty food stimuli employed in this study originated from a dataset by Kroemer et al. ^9^. The picture selection was based on the following criteria: (1) Social stimuli needed to be free of eating disorder relevant content (e.g. pictures showing women in bikini were excluded); (2) Only neutral and social stimuli with valence and arousal ratings that were similar in AN patients and HC (established in an independent pilot study) were selected; (3) The stimuli of all three conditions had to be similar regarding the entropy of intensity distribution and brightness to exclude that the perception is biased by differing stimulus properties. Neutral (arousal=2.6(0.5)) and social stimuli (arousal=4.5(0.9)) presented in the task differed significantly regarding their arousal (T(44.24)=10.64; p<0.001).

**SM 1.4. FMRI quality control**

We evaluated the quality of the fMRI data by manual inspection and using artifact detection tools (ART) ^10^. Volumes that exceeded an intensity threshold of three standard deviations or a threshold of 2mm normalized movement in any direction were classified as outliers.

**Supplementary table 2**

*Means and Standard Deviation of intensity and motion-outlier in both samples*

|  | Sample | | Statistics | |
| --- | --- | --- | --- | --- |
|  | acAN | HC_acAN_ | T | P |
|  | *recAN* | *HC_recAN_* |
| Intensity-outlier | 6.87±3.87 | 8.23±4.89 | -1.26 | 0.212 |
|  | *7.18±5.00* | *7.51±5.59* | *-0.26* | *0.794* |
| Motion-outlier | 3.31±7.05 | 10.14±22.43 | -1.73 | 0.092 |
|  | *7.51±4.95* | *7.52±5.59* | *-1.41* | *0.158* |

*Note*. acAN = acute anorexia nervosa patients, recAN = recovered anorexia nervosa patients; HC_acAN_ = healthy subsample age-matched to acute anorexia nervosa patients, HC_recAN_ = healthy subsample age-matched to recovered anorexia nervosa patients.

**SM 1.5 Cortical thickness measurement**

Surface reconstruction was conducted for each hemisphere including tessellation of the gray matter-white matter boundary, automated topology correction, and surface deformation following intensity gradients to optimally place the gray-white and gray-cerebrospinal fluid borders at the location where the greatest shift in intensity defines the transition to the other tissue class. Based on the resulting surfaces, cortical thickness were calculated at each vertex as the closest distance from the gray-white boundary to the pial surface. Segmentation and surface reconstruction quality were assured by manual inspection of all raw MRI volumes, segmented volumes in three planes and pial as well as inflated volumes. Possible misclassification of brain tissue was resolved manually by providing points of the tissue boundaries via the graphical user interface tkmedit. After rerunning the reconstruction procedure, which uses the information given in tkmedit, the quality of the result was reassessed. Mean cortical thickness values of the region of interest were extracted and subjected to SPM 8 to implement as a covariable.

**SM 2 Results – GLM**

Stimulus-specific activity was found both in the subliminal (sublim) and supraliminal (supra) stimulation conditions, demonstrating that our task worked as intended. In detail, the contrasts social_supra_>neutral_supra_ (T=8.87; *p_FWE_*<0.05; Figure SM2) and social_sublim_>neutral_sublim_ (T=9.06; *p_FWE_*<0.05; Figure 2C) showed increased activation in regions associated with reward processing, including the ventral striatum, anterior cingulate cortex and insula. When investigating the contrast food_supra_>neutral_supra_ (T=6.81; *p_FWE_*<0.05; Figure 2B), we found increased activity at clusters known to be involved in food processing ^11^, e.g. posterior fusiform gyrus, while no cluster was found in the contrast food_sublim_>neutral_sublim_ .

**Supplementary figure 1**

*Test of stimulus type specific activation patterns under subliminal and supraliminal stimulation condition.*

*
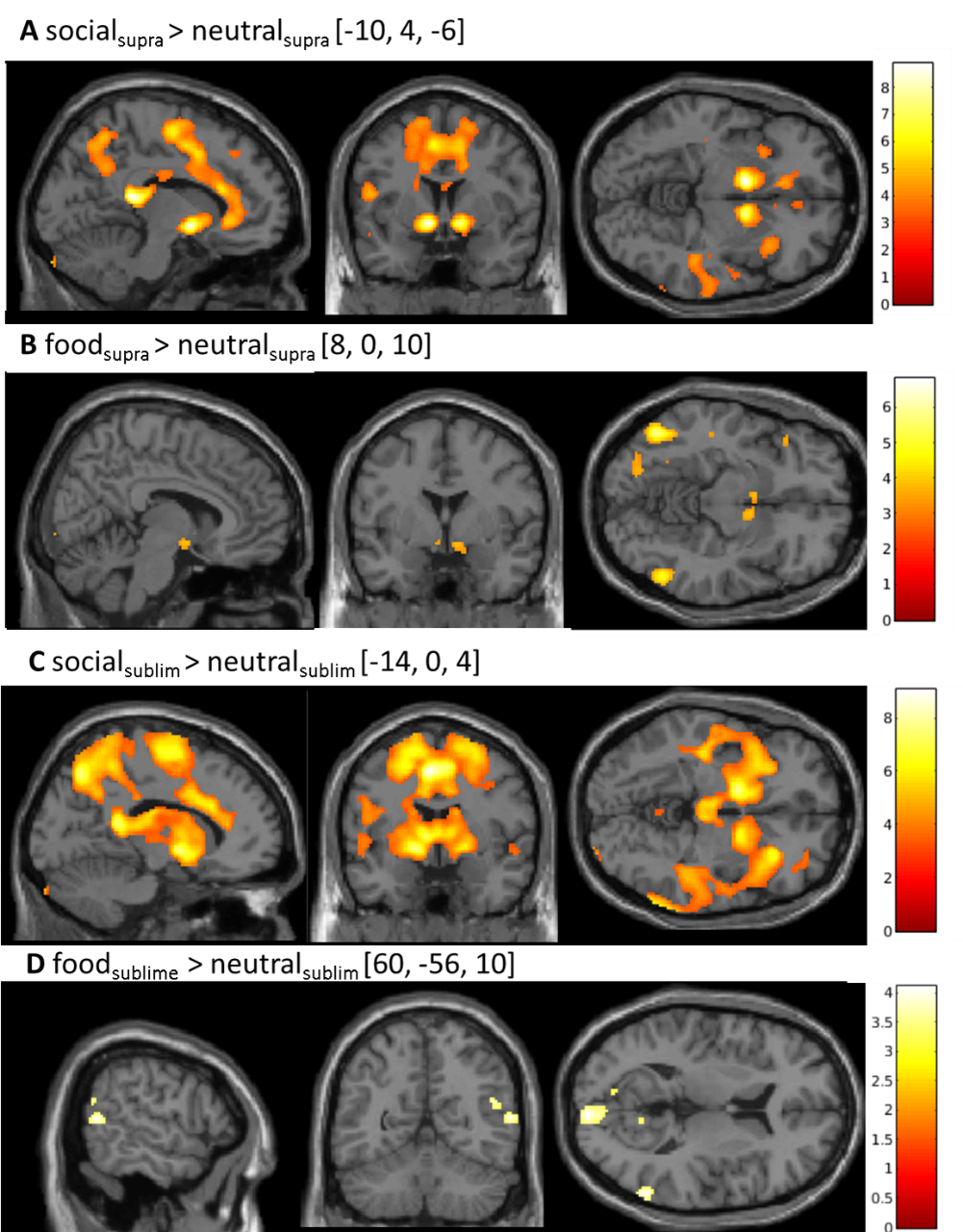
*

*Note*. Brain maps are displayed at p=0.001; Coordinates in Montreal Neurological Institute (MNI) space [x, y, z]; color bars represent t-values.

**SM 3 Results - MVPA**

**SM 3.1 Main effects of classification contrasts**

Above chance accuracy of the classification contrast food vs. neutral and social vs. neutral was located in secondary visual areas for the acAN vs. HC_acAN_ sample and the recAN vs. HC_recAN_ sample.

**Supplementary figure 2**

*Above chance accuracy of the classification contrasts of interest*


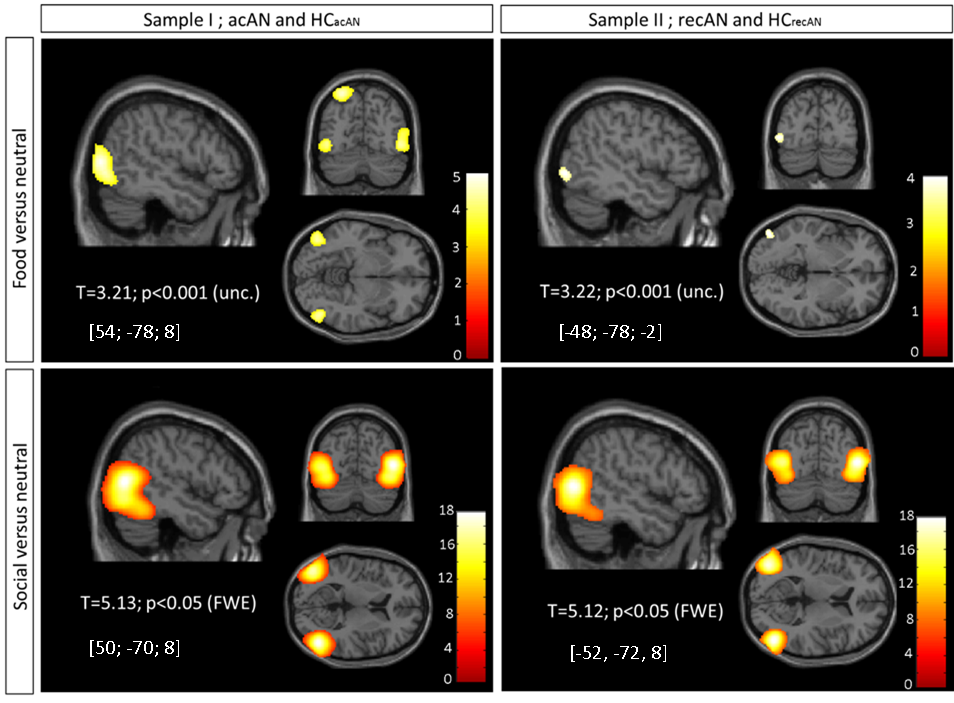


*Note*. acAN = acute anorexia nervosa patients, recAN = recovered anorexia nervosa patients; HC_acAN_ = healthy subsample age-matched to acute anorexia nervosa patients, HC_recAN_ = healthy subsample age-matched to recovered anorexia nervosa patients.

**SM 3.2 Cortical thickness and comorbidities as covariates**

When controlling for cortical thickness (acAN: mean (SD)=2.45 (0.51); HC: mean (SD)=2.74 (0.50)) and comorbid disorder (classified as yes or no), the group comparison of the classification contrast food vs. neutral stimuli remained significant.

**Supplementary figure 3**

Group difference in classification accuracy between acAN and HC_acAN_ in the food vs. neutral contrast (p<0.05, FWE) controlled for cortical thickness.


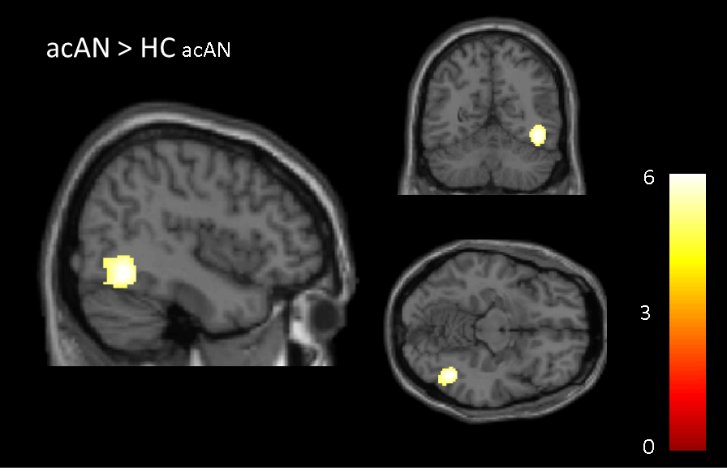


**Supplementary figure 4**

Group difference in classification accuracy between acAN and HC_acAN_ in the food vs. neutral contrast (p<0.05, FWE) controlled for comorbid disorders.


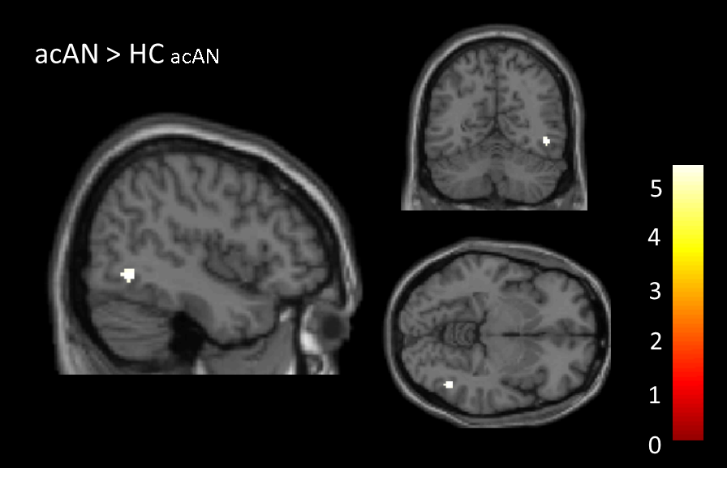


**SM 4 Bayesian independent sample t-test**

In order to investigate processes of normalization between recAN and HC, we tested the evidence to support the null hypothesis (no group difference). In order to do so, we calculated Bayesian independent samples t-test using extracted β-values of posterior FG that were previously established to be different between acutely ill acAN patients and HC according to the MVPA approach. The t-test was calculated with a prior described by a Cauchy distribution with a width parameter of 0.71 (default).

**Supplementary table 3**

Results of the Bayesian independent sample t-test.

| Cluster | BF₀₁ | Error % |
| --- | --- | --- |
| Posterior FG | 8.53 | -4.35e-4 |

**References**

1. Fichter, M. & Quadflieg, N. *SIAB. Struckturiertes Inventar fuer anorektische und Bulimische Essstoerungen nach DSM-IV und ICD-10*. (Huber, 1999).

2. Munkres, J. Algorithms for the assignment and transportation problems. *J. Soc. Ind. Appl. Math.* **5**, 32–38 (1957).

3. Harris, P. A. *et al.* Research electronic data capture (REDCap)—a metadata-driven methodology and workflow process for providing translational research informatics support. *J. Biomed. Inform.* **42**, 377–381 (2009).

4. Boehm, I. *et al.* Subliminal and supraliminal processing of reward-related stimuli in anorexia nervosa. *Psychol. Med.* 1–11 (2017) doi:10.1017/S0033291717002161.

5. Von Aster, M., Neubauer, A. & Horn, R. Wechsler Intelligenztest für Erwachsene (WIE). Deutschsprachige Bearbeitung und Adaptation des WAIS-III von David Wechsler. *FrankfurtMain Ger. Harcourt Test Serv.* (2006).

6. Daseking, M., Petermann, U. & Petermann, F. Intelligenzdiagnostik mit dem HAWIK-IV. *Kindh. Entwickl.* **16**, 250–259 (2007).

7. Lang, P. J., Bradley, M. M. & Cuthbert, B. N. *International affective picture system (IAPS): Instruction manual and affective ratings*. vol. 2 (Center of Research in Psychophysiology, University of Florida, 1999).

8. Wessa, M. *et al.* EmoPicS: Subjektive und psychophysiologische Evaluation neuen Bildmaterials für die klinisch-biopsychologische Forschung. *Available Michele Wessa Michele Wessa Med Uni-Heidelb. De* (2010).

9. Kroemer, N. B. *et al.* (Still) longing for food: Insulin reactivity modulates response to food pictures. *Hum. Brain Mapp.* **34**, 2367–2380 (2013).

10. Whitfield-Gabrieli, S. *et al.* Hyperactivity and hyperconnectivity of the default network in schizophrenia and in first-degree relatives of persons with schizophrenia. *Proc. Natl. Acad. Sci.* **106**, 1279–1284 (2009).

11. van der Laan, L. N., de Ridder, D. T. D., Viergever, M. A. & Smeets, P. A. M. The first taste is always with the eyes: A meta-analysis on the neural correlates of processing visual food cues. *NeuroImage* **55**, 296–303 (2011).
